# Chlorogenic Acid Attenuates Oxidative Stress-Induced Intestinal Mucosa Disruption in Weaned Pigs

**DOI:** 10.3389/fvets.2022.806253

**Published:** 2022-02-14

**Authors:** Jiali Chen, Daiwen Chen, Bing Yu, Yuheng Luo, Ping Zheng, Xiangbing Mao, Jie Yu, Junqiu Luo, Zhiqing Huang, Hui Yan, Jun He

**Affiliations:** ^1^Institute of Animal Nutrition, Sichuan Agricultural University, Chengdu, China; ^2^Key Laboratory of Animal Disease-Resistance Nutrition, Chengdu, China; ^3^Shandong Provincial Key Laboratory of Animal Biotechnology and Disease Control and Prevention, Department of Animal Science and Veterinary Medicine, Shandong Agricultural University, Tai'an, China

**Keywords:** antioxidant capacity, intestinal barrier function, natural polyphenolic, small intestine, swine

## Abstract

Chlorogenic acid (CGA) is a natural polyphenol that possesses potent antioxidant activity. However, little is known about its exact role in regulating the intestinal health under oxidative stress. This study was conducted to explore the effect of dietary CGA supplementation on intestinal barrier functions in weaned pigs upon oxidative stress. Twenty-four weaned pigs were allocated to three treatments and were given a basal diet (control) or basal diet containing CGA (1,000 mg/kg) for 21 days. Pigs were challenged by sterile saline (control) or diquat [10 mg/kg body weight (BW)] on the 15th day. Results showed that CGA attenuated the BW reduction, reduced the serum concentrations of diamine oxidase and D-lactate, and elevated serum antioxidant enzymes activities in diquat-challenged weaned pigs (*P* < 0.05). Moreover, diquat challenge decreased villus height and activities of sucrase and alkaline phosphatase in jejunum and ileum (*P* < 0.05), but CGA elevated the villus height and enzyme activities in the intestinal mucosa (*P* < 0.05). In addition, CGA not only decreased the expression levels of Bax, caspase-3, and caspase-9 (*P* < 0.05) but also elevated the expression levels of sodium glucose transport protein-1, glucose transporter-2, occludin, claudin-1, zonula occludens-1, and antioxidant genes such as nuclear factor erythroid-derived 2-related factor 2 and heme oxygenase-1 in intestinal mucosa of weaned pigs upon oxidative stress (*P* < 0.05). These findings suggested that CGA can attenuate oxidative stress-induced growth retardation and intestinal mucosa disruption, which was linked to elevated antioxidative capacity and enhanced intestinal barrier integrity.

## Introduction

The intestinal mucosa plays as one of the key barriers in living organisms. It is mainly composed of intestinal epithelial cells and intercellular tight junctions of enterocytes, which not only plays a critical role in nutrient absorption but also constitutes the first line of defense against various pathogens and toxins in the intestinal lumen (1, 2). However, a variety of stimuli such as malnutrition, bacterial infections, and stresses can impair the integrity of the intestinal epithelium. In particular, oxidative stress, as a result of reactive oxygen species (ROS) overexpression, is a common feature of many chronic and acute intestinal diseases (3). For instance, it can induce atrophy of the intestinal mucosa and lead to the reduction of intestinal digestion and absorption ability (3). Moreover, oxidative stress-induced overproduction of ROS could also induce DNA damage and apoptosis of the intestinal epithelial cells, which increased intestinal permeability and facilitates translocation of luminal antigens into sub-epithelial tissues, leading to growth retardation and a series of intestinal and systemic diseases (4, 5). Therefore, the potential interventions to alleviate the intestinal oxidative stress have attracted considerable research interest worldwide.

A variety of polyphenols have previously been reported to have the potential to alleviate oxidative stress-induced diseases (6). Chlorogenic acid (CGA), formed by esterification of caffeic acid and quinic acid, is one of the most abundant natural polyphenolic compounds presented in coffee, fruits, and vegetables (7). Previous studies indicated that CGA has multiple benefits for mammalian animals, including antioxidant, anti-inflammatory, and antibacterial activities (8, 9). CGA has been reported to alleviate intestinal ischaemia and reperfusion injury by improving the antioxidant activities and ameliorate endotoxin-induced intestinal injury in rats (10, 11). These attributes should make it an attractive health product for various oxidative stress-induced injuries and diseases. In a previous study, CGA was found to improve growth performance and intestinal health through elevating the activities of antioxidant enzymes in weaned pigs (12). However, there is little information available in the scientific literature on the evaluation of the protective effect and potential mechanisms of CGA supplementation on oxidative stress-induced intestinal injury in weaned pigs.

In the present study, we explored the effects of dietary supplementation with CGA on growth performance, antioxidant capacity, and the structure and functions of intestinal mucosa in pigs' exposure to oxidative stress. The oxidative stress was induced by using diquat that was a classic agent for the construction of oxidative stress models (13, 14). This study will assist in developing of CGA to attenuate various oxidative stress-induced injury or diseases.

## Materials and Methods

### Animals, Diet, and Experimental Design

Twenty-four weaned pigs (Duroc × Landrace × Yorkshire, 21 days of age), with an initial average body weight (BW) of 7.47 ± 0.50 kg, were randomly allotted to one of three treatments (eight replicates each treatment) in a completely randomized design for a 21-day study. The three experimental treatments were as follows: (1) non-challenged weaned pigs fed a basal diet [control (CON) group]; (2) diquat-challenged weaned pigs fed a basal diet (Diquat group); and (3) diquat-challenged weaned pigs fed a basal diet supplemented with CGA (1,000 mg/kg; DCGA group). The supplementary level of CGA to feed was based on our previous study (12). On the 15th day of the trial, pigs from the CON group were intraperitoneally injected with sterile saline. The other two groups were challenged by diquat (intraperitoneal injection, diquat dibromide monohydrate, PS365; Sigma Co.) at a dose of 10 mg/kg BW. The basal diet was formulated according to our previous study (12), and ingredient composition and nutrient level are presented in Supplementary Table S1. All weaned pigs were housed in individual metabolic cages (1.5 × 0.7 × 1.0 m) and had free access to fresh water and feed throughout the experiment. The room temperature was maintained at 25–28°C, and relative humidity controlled at 55–65%. The feed intake per pig was recorded daily, and pigs were weighed on the mornings of days 14 and 21 of the experiment. The average daily weight gain (ADG), average daily feed intake (ADFI), and feed conversion (F/G) were calculated.

### Sample Collection

At the end of the trial, blood samples were collected *via* jugular venipuncture from all weaned pigs after 12-h fasting. The serum samples were obtained by centrifugation at 3,000 × g for 15 min at 4°C and then stored at −20°C for further analysis. Subsequently, pigs were euthanized with an intravenous injection of sodium pentobarbital (200 mg/kg BW), and the abdomen was immediately opened to collect the intestinal segments (duodenum, jejunum, and ileum) according to the method described by Zheng et al. (15). About 3-cm segments of the middle of duodenum, jejunum, and ileum were isolated, gently flushed with ice-cold phosphate-buffered saline (PBS), and then fixed in 4% paraformaldehyde solution for histological analyses. The mucosa samples were harvested by scraping the segment using a sterile glass slide, snap-frozen in liquid nitrogen, and then stored at −80°C until further analysis.

### Determination of Serum Parameters

The superoxide dismutase (SOD), catalase (CAT), glutathione peroxidase (GSH-Px), total antioxidant capacity (T-AOC), malondialdehyde (MDA), diamine oxidase (DAO), and D-lactic acid were measured by using the assay kits (Nanjing Jiancheng Institute of Bioengineering, Jiangsu, China) according to the manufacturer's instructions.

### Intestinal Morphology Analysis

After fixation with 4% paraformaldehyde solution, the intestinal morphology was estimated according to previous method (16). Briefly, the duodenum, jejunum, and ileum segments were dehydrated in a graded ethanol series, embedded in paraffin, sliced into 5-μm-thick cross-sections by using a microtome and stained with hematoxylin and eosin. A minimum of 10 well-orientated crypt-villus units from each intestinal segment were chosen and measured. Villus height and crypt depth were determined with image processing and analysis system (ImagePro Plus 6.0, Media Cybernetics, Inc., Rockville, MD, USA). The ratio of villus height to crypt depth (VCR) was calculated as the villus height divided by crypt depth.

### Intestinal Mucosa Parameters

About 1 g of mucosa samples of each section were homogenized after dilution with ice-cold saline solution (1:9, w/v) and then centrifuged (2,500 × g, 10 min, 4°C) to collect the supernatant. The activities of lactase, sucrose, maltase, and alkaline phosphatase (AKP) in duodenal, jejunal, and ileal mucosa were measured by using the assay kits (Nanjing Jiancheng Institute of Bioengineering, Jiangsu, China) according to the manufacturer's instructions. The total protein content in the mucosa samples was determined using the Bradford brilliant blue method. All samples were measured in triplicate. Enzyme activities were presented as units (U) per milligram of protein.

### RNA Isolation and qPCR

Total RNA of duodenum, jejunum, and ileum mucosa was isolated by using TRIzol Reagent (TaKaRa, Dalian, China). The RNA concentration and purity were assayed by spectrophotometer (Beckman Coulter, DU800) at 260 and 280 nm. For each sample, reverse transcription was performed using the PrimeScript RT reagent kit with gDNA Eraser (TaKaRa, Dalian, China) according to the manufacturer's instructions. All primers were synthesized commercially by Sangon Biotech Limited and were shown in Supplementary Table S2. Quantitative real-time PCR was performed to analyze the expression levels of sodium glucose transport protein-1 (SGLT1), glucose transporter-2 (GLUT2), zonula occludens-1 (ZO-1), occludin, claudin-1, B-cell lymphoma-2–associated X protein (Bax), B-cell lymphoma-2 (Bcl-2), caspase-3, caspase-9, nuclear factor erythroid-derived 2-related factor 2 (Nrf2), Kelch-like epichlorohydrin–associated protein 1 (Keap1), and heme oxygenase-1 (HO-1) using SYBR® Premix Ex TaqTM II (Tli RNaseH Plus) reagents (TakaRa, Dalian, China) and the QuanStudioTM 6 Flex Real-Time PCR detection system (Applied Biosystems, Foster City, CA, USA). Each reaction was performed in a 10-μl reaction volume, which contained 5 μl of SYBR Premix Ex TaqTM (2 ×), 1 μl of each primer, 2 μl of doubled-distilled water, and 1 μl of cDNA template. The PCR cycling parameters were as follows: 95°C for 30 s, 40 cycles of 95°C for 10 s, 60°C for 25 s, and 72°C for 5 min. After amplification, melting curve analysis was performed after each real-time quantitative PCR assay to verify the specificity of the reactions. The glyceraldehyde-3-phosphate dehydrogenase (GAPDH) gene was used as the reference gene. The target gene mRNA expression level was calculated using the 2^−ΔΔCt^ method (15). Each sample was tested simultaneously in triplicate on the same PCR plate.

### Statistical Analysis

Individual pig was used as the experimental unit, and all data were analyzed by general liner mode (GLM) procedure of SAS 9.4 (SAS Institute Inc., Cary, NC, USA) after being assessed for normal distribution using the Shapiro–Wilk's statistic (W > 0.05). Statistical differences among groups were determined by Tukey's multiple-range test. Results were presented as means and SEMs. Differences were taken to indicate significance when *P* < 0.05.

## Results

### Growth Performance

The growth performance was not significantly affected by CGA before diquat challenge (1–14 days) (Table 1). However, diquat challenge resulted in a 44.42% reduction of ADFI and a 86.18% decrease of ADG compared with the CON pigs (*P* < 0.05). In contrast, dietary supplementation with CGA (1,000 mg/kg) suppressed the diquat-induced decreases in ADFI (*P* < 0.05). Besides, dietary CGA supplementation attenuated the decrease of ADG in pigs upon diquat challenge (*P* < 0.05) although the ADG of pigs in DCGA group was still lower than that in the CON group (*P* < 0.05).

**Table 1:** Effects of CGA on the growth performance in weaned pigs upon oxidative stress^1^.

**Items**	**CON**	**Diquat**	**DCGA**	**SEM**	***P***
**1–14 days**					
ADFI (g)	385.27	386.13	398.24	19.67	0.964
ADG (g)	261.61	266.79	294.40	11.99	0.547
F/G	1.47	1.49	1.37	0.06	0.773
**15–21 days**					
ADFI (g)	540.33^a^	300.27^b^	428.19^ab^	34.86	0.007
ADG (g)	361.67^a^	50.00^c^	224.17^b^	35.57	<0.001
F/G	n/a	n/a	n/a	–	–

*^1^Results are presented as mean and SEM. Labeled means in a row without a common superscript letter differ, P < 0.05. ADFI, average daily feed intake; ADG, average daily gain; F/G, feed efficiency (the ratio of ADFI and ADG); CON, piglets fed a basal diet; Diquat, piglets fed the basal diet and challenged by diquat; DCGA, piglets fed the basal diet containing CGA (1,000 mg/kg) and challenged by diquat*.

### Serum Parameters

**Figure 1:**
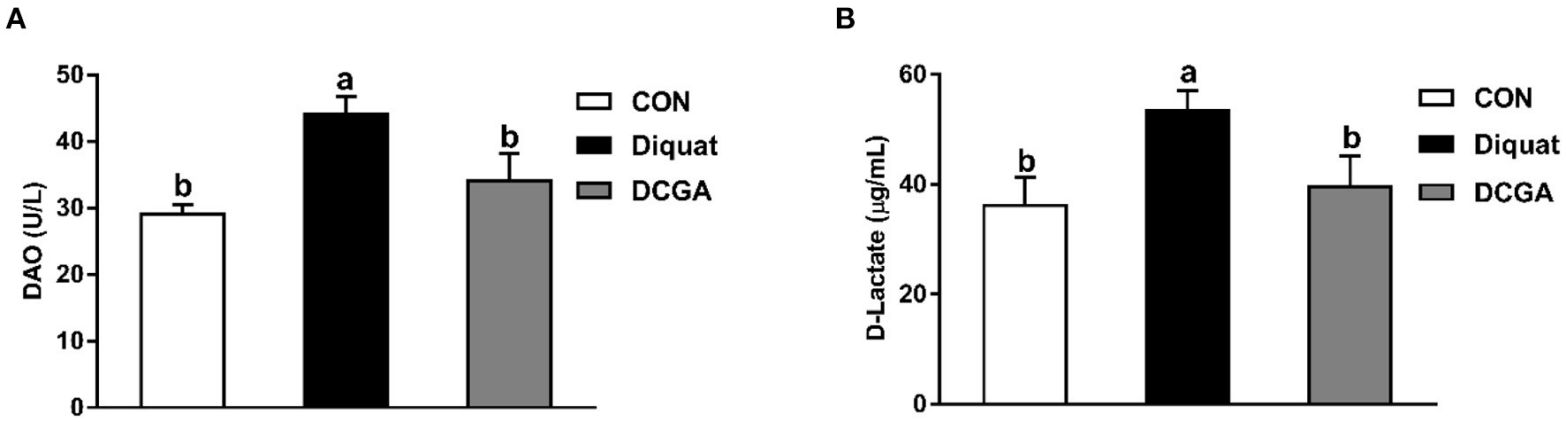
Effects of CGA on serum DAO **(A)** and D-Lactate **(B)** levels in piglets upon oxidative stress. DAO, diamine oxidase; CON, piglets fed a basal diet; Diquat, piglets fed the basal diet and challenged by diquat; DCGA, piglets fed the basal diet containing CGA (1,000 mg/kg) and challenged by diquat.

As shown in Figure 1 and Table 2, diquat-challenged pigs showed a significant increase in serum levels of DAO, D-lactate, and MDA (*P* < 0.05), while a significant decrease in serum activities of SOD and GSH-Px (*P* < 0.05). However, dietary CGA supplementation attenuated the increase of DAO and D-lactate levels (*P* < 0.05) and the decrease of GSH-Px and SOD activities (*P* < 0.05) in the serum to levels observed in pigs of CON group. Meanwhile, CGA supplementation suppressed the diquat-induced increase of serum MDA concentration but still higher than that in the CON group (*P* < 0.05). There were no significant differences in the levels of CAT and T-AOC among the groups (*P* > 0.05).

**Table 2:** Effects of CGA on serum antioxidant parameters in weaned pigs upon oxidative stress^1^.

**Item**	**CON**	**Diquat**	**DCGA**	**SEM**	***P***
SOD (U/ml)	77.84^a^	56.31^b^	76.80^a^	8.19	<0.0001
MDA (nmol/ml)	3.40^c^	11.94^a^	7.53^b^	0.94	<0.0001
GSH-Px (U/ml)	411.04^a^	272.05^b^	387.88^a^	32.39	<0.0001
CAT (U/ml)	11.06	10.10	14.08	1.07	0.302
T-AOC (U/ml)	5.96	5.76	5.51	0.27	0.812

*^1^Results are presented as mean and SEM. Labeled means in a row without a common superscript letter differ, P < 0.05. GSH-Px, glutathione peroxidase; CAT, catalase; T-AOC, total antioxidant capacity; SOD, superoxide dismutase; MDA, malondialdehyde; CON, piglets fed a basal diet; Diquat, piglets fed the basal diet and challenged by diquat; DCGA, piglets fed the basal diet containing CGA (1,000 mg/kg) and challenged by diquat*.

### Intestinal Morphology

**Figure 2:**
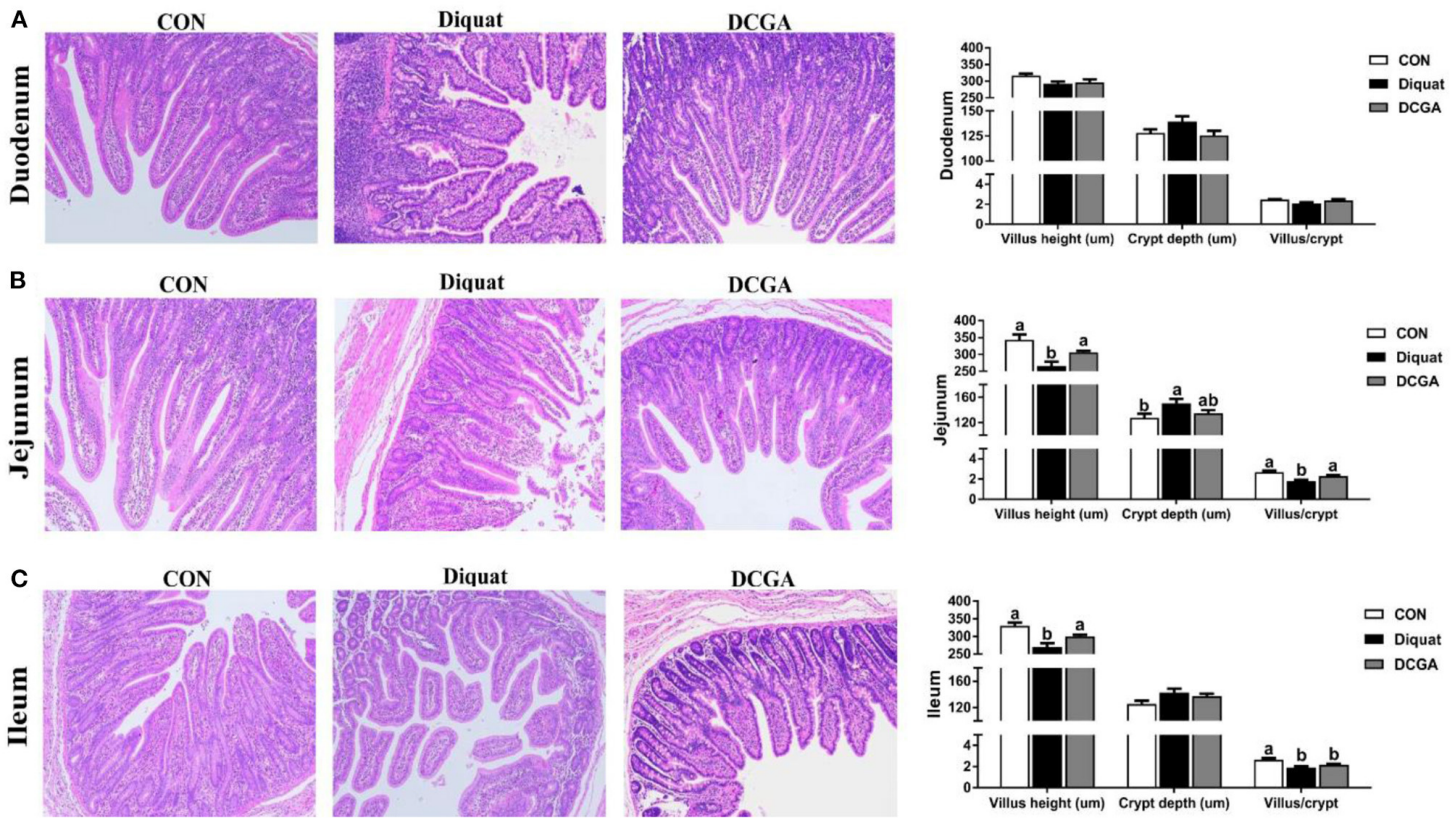
Effects of CGA on intestinal morphology in the Duodenum **(A)**, Jejunum **(B)**, and Ileum **(C)** of piglets upon oxidative stress. CON, piglets fed a basal diet; diquat, piglets fed the basal diet and challenged by diquat; DCGA, piglets fed the basal diet containing CGA (1,000 mg/kg) and challenged by diquat.

As shown in Figure 2, compared with the CON group, diquat-challenged pigs showed a significant decrease in villus height and the VCR in jejunum and ileum (*P* < 0.05), while a significant increase in crypt depth in jejunum (*P* < 0.05). Compared with pigs in the Diquat group, pigs in the DCGA group exhibited increased villus height and VCR in jejunum as well as villus height in ileum to the levels observed in pigs of the CON group (*P* < 0.05). However, the ileal VCR of pigs in DCGA group was still lower than that in the CON group.

### Enzyme Activities and the mRNA Expression Levels of Digestion and Absorption-Related Genes

**Figure 3:**
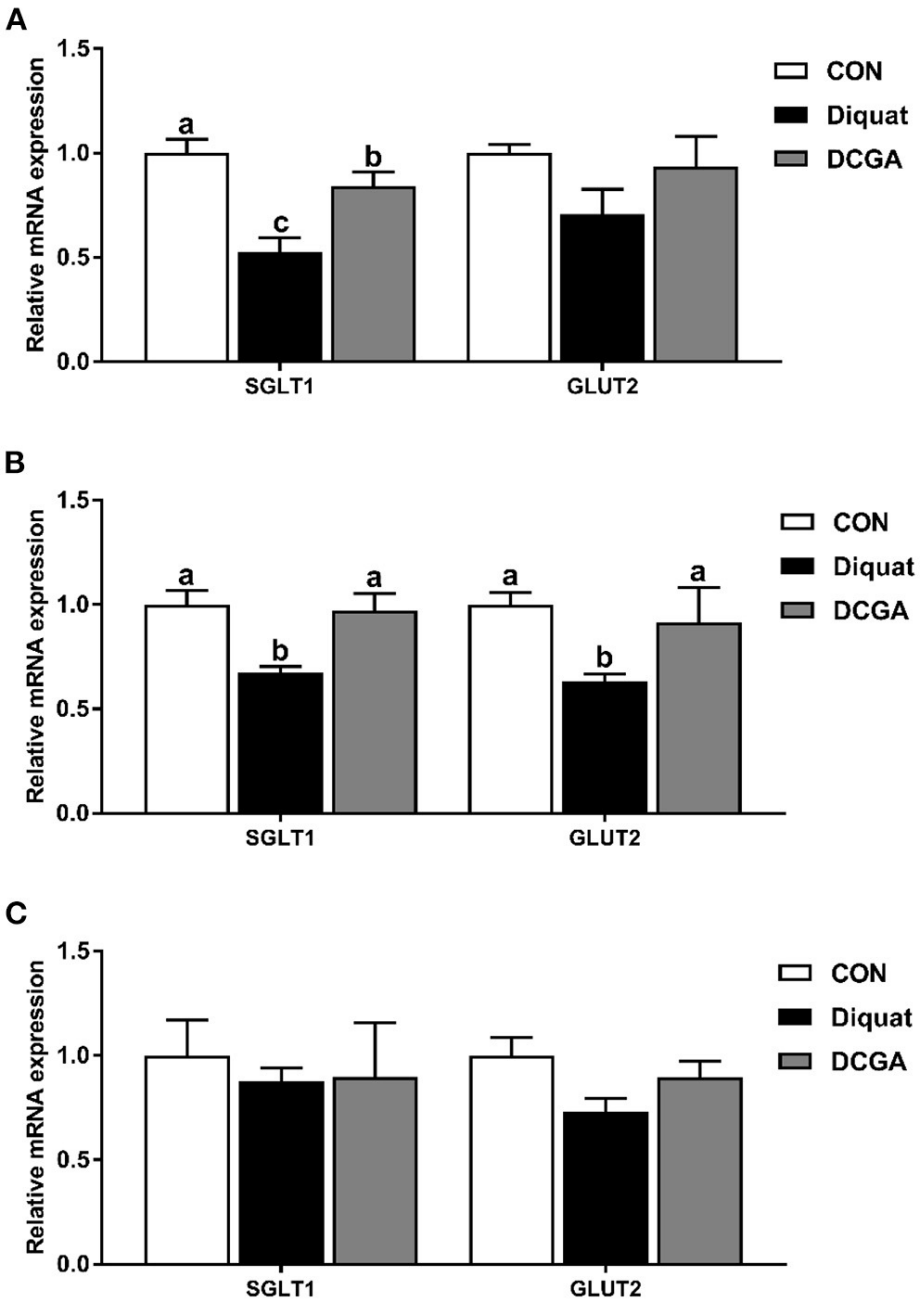
Effects of CGA on expression levels of intestinal digestion and absorption-related genes in the Duodenum **(A)**, Jejunum **(B)**, and Ileum **(C)**. SGLT1, sodium glucose transport protein-1; GLUT2, glucose transporter-2; CON, piglets fed a basal diet; Diquat, piglets fed the basal diet and challenged by diquat; DCGA, piglets fed the basal diet containing CGA (1,000 mg/kg) and challenged by diquat. ^a, b^ Bars with different letters between treatment groups indicate significant differences (*P* < 0.05).

As shown in Table 3, diquat challenge not only decreased the mucosal activities of disaccharidases such as the sucrase, lactase, and maltase in the duodenum and jejunum (*P* < 0.05) but also decreased the mucosal activity of AKP in the jejunum and ileum (*P* < 0.05). However, compared with pigs in the Diquat group, dietary CGA supplementation significantly increased the activities of sucrose, lactase, and maltase in duodenum; the activities of sucrase, maltase, and AKP in jejunum; and the activities of sucrase and AKP in ileum (*P* < 0.05). The lactase activity of duodenum and jejunum as well as the sucrase activity of ileum in pigs of DCGA group were still lower than those in the CON group (*P* < 0.05). Meanwhile, lower mRNA expression levels of SGLT1 in duodenum as well as SGLT1 and GLUT2 in jejunum were observed in pigs of Diquat group (Figure 3). However, dietary CGA supplementation mitigated the negative effects of diquat (*P* < 0.05).

**Table 3:** Effects of CGA on intestinal enzyme activities in weaned pigs upon oxidative stress^1^.

**Item**	**CON**	**Diquat**	**DCGA**	**SEM**	***P***
**Duodenum**					
Sucrase (U/mg prot)	75.37^a^	41.63^b^	65.71^a^	4.83	0.007
Lactase (U/mg prot)	60.01^a^	30.80^c^	45.09^b^	4.93	0.020
Maltase (U/mg prot)	259.11^a^	206.07^b^	257.16^a^	9.25	0.018
AKP (U/mg prot)	60.66	54.42	64.42	4.76	0.713
**Jejunum**					
Sucrase (U/mg prot)	185.81^a^	97.88^b^	154.17^a^	10.70	<0.0001
Lactase (U/mg prot)	77.88^a^	41.84^c^	61.32^b^	4.88	0.002
Maltase (U/mg prot)	265.75^a^	193.79^b^	267.38^a^	10.60	0.001
AKP (U/mg prot)	38.08^a^	22.60^b^	40.84^a^	2.30	<0.0001
**Ileum**					
Sucrase (U/mg prot)	121.87^a^	56.14^c^	95.29^b^	10.37	0.004
Lactase (U/mg prot)	65.45	57.92	63.86	2.96	0.584
Maltase (U/mg prot)	236.62	212.72	240.66	9.63	0.468
AKP (U/mg prot)	54.81^a^	32.21^b^	48.74^a^	2.67	<0.0001

*^1^Results are presented as mean and SEM. Labeled means in a row without a common superscript letter differ, P < 0.05. AKP, alkaline phosphatase; CON, piglets fed a basal diet; Diquat, piglets fed the basal diet and challenged by diquat; DCGA, piglets fed the basal diet containing CGA (1,000 mg/kg) and challenged by diquat*.

### mRNA Expression Levels of Intestinal Function and Barrier-Related Genes

**Figure 4:**
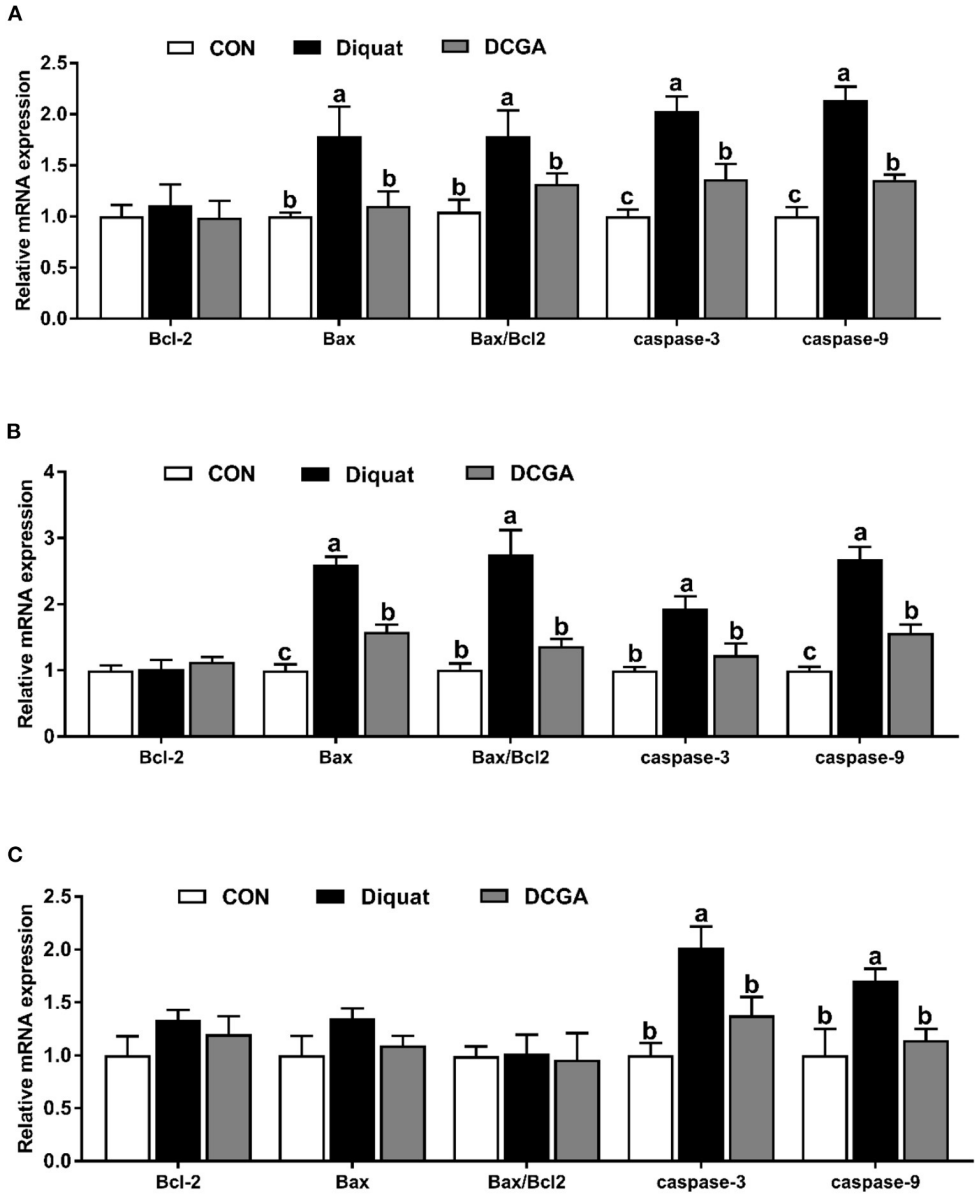
Effects of CGA on expression levels of intestinal barrier-related genes in the Duodenum **(A)**, Jejunum **(B)**, and Ileum **(C)**. ZO-1, zonula occludens 1; CON, piglets fed a basal diet; Diquat, piglets fed the basal diet and challenged by diquat; DCGA, piglets fed the basal diet containing CGA (1,000 mg/kg) and challenged by diquat. ^a, b^ Bars with different letters between treatment groups indicate significant differences (*P* < 0.05).

**Figure 5:**
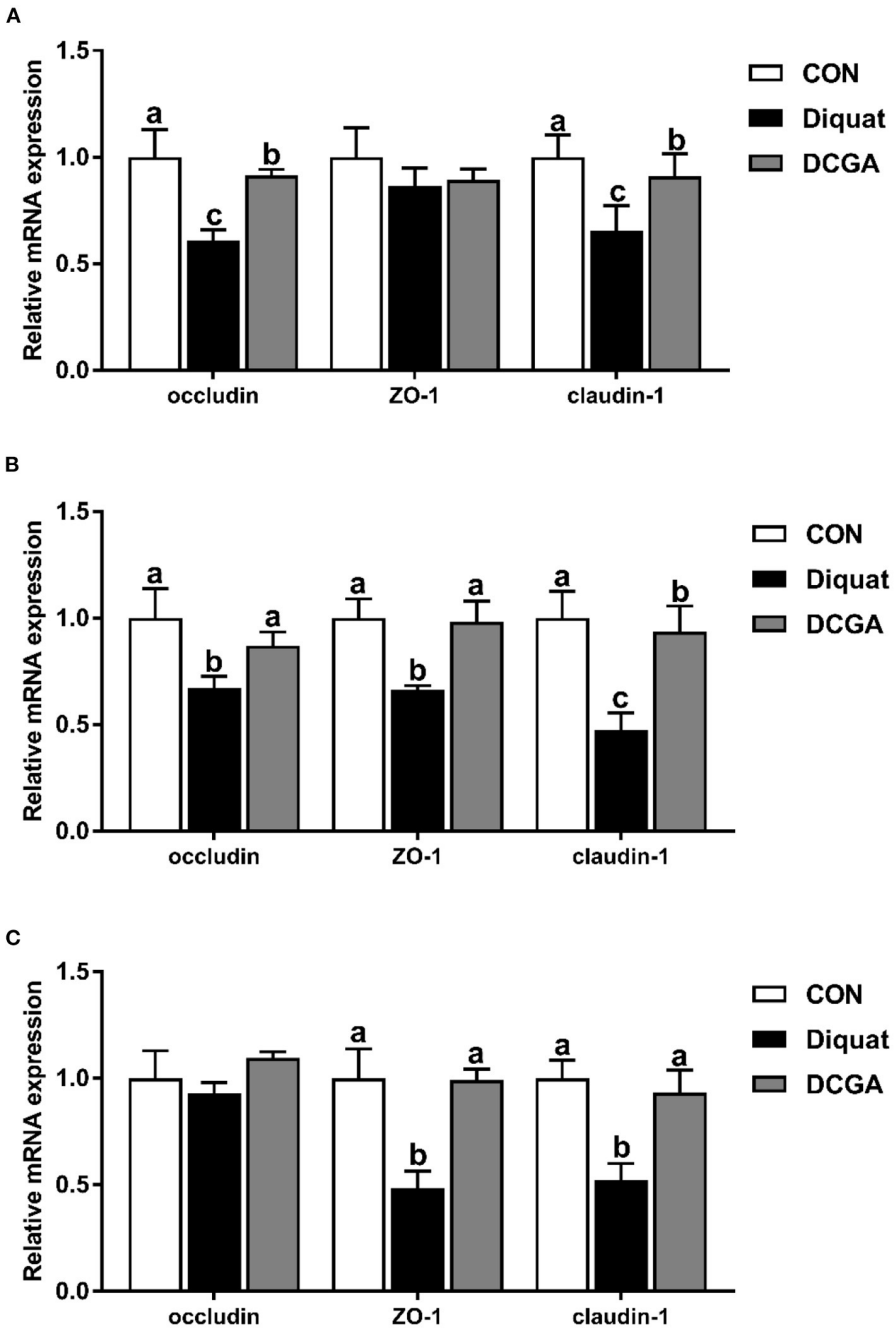
Effects of CGA on expression levels of apoptosis-related genes in the Duodenum **(A)**, Jejunum **(B)**, and ileum **(C)**. Bcl2, B-cell lymphoma-2; Bax, B-cell lymphoma-2–associated X protein; CON, piglets fed a basal diet; Diquat, piglets fed the basal diet and challenged by diquat; DCGA, piglets fed the basal diet containing CGA (1,000 mg/kg) and challenged by diquat. ^a, b^ Bars with different letters between treatment groups indicate significant differences (*P* < 0.05).

As presented in Figure 4, diquat strongly increased the mRNA expression levels of Bax and Bax/Bcl2 in the duodenum and jejunum as well as the mRNA expression levels of caspase-3 and caspase-9 in the three intestinal sections. However, dietary CGA supplementation mitigated the negative effects of diquat (*P* < 0.05), although the caspase-3 mRNA expression levels in duodenum and the caspase-9 mRNA expression levels in duodenum and jejunum were still higher than those in the group (*P* < 0.05). In addition, a significant decrease in mRNA expression levels of occludin in duodenum and jejunum, ZO-1 in jejunum and ileum, and claudin-1 in the three intestinal sections was observed under diquat stimulation (Figure 5), which was strongly inhibited by CGA (*P* < 0.05). However, the mRNA expression levels of claudin-1 in duodenum and jejunum of pigs in DCGA group were still higher than those in the CON group (*P* < 0.05).

### mRNA Expression Levels of Critical Molecules in Antioxidant Signaling Pathway

**Figure 6:**
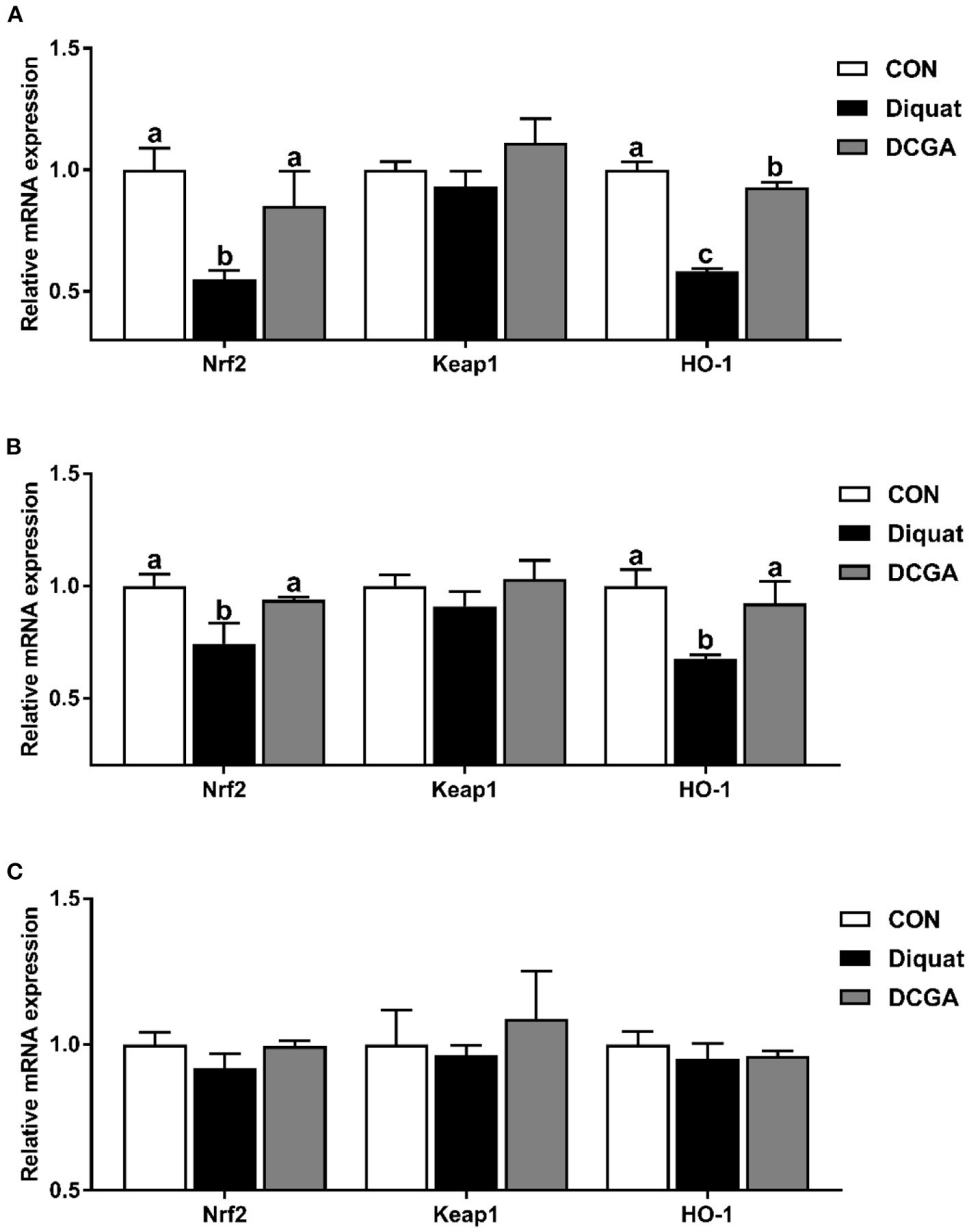
Effects of CGA on expression levels of intestinal antioxidant-related key genes in the Duodenum **(A)**, Jejunum **(B)**, and Ileum **(C)**. Nrf2, nuclear factor erythroid-derived 2-related factor 2; Keap1, Kelch-like epichlorohydrin–associated protein 1; HO-1, heme oxygenase-1; CON, piglets fed a basal diet; Diquat, piglets fed the basal diet and challenged by diquat; DCGA, piglets fed the basal diet containing CGA (1,000 mg/kg) and challenged by diquat. ^a, b^ Bars with different letters between treatment groups indicate significant differences (*P* < 0.05).

As shown in Figure 6, diquat challenge significantly decreased the expression levels of Nrf2 and HO-1 in duodenum and jejunum (*P* < 0.05). However, dietary CGA supplementation significantly suppressed the diquat-induced decrease in the expression levels of Nrf2 and HO-1 (*P* < 0.05). Moreover, duodenal HO-1 mRNA expression level of pigs was still lower in DCGA group than that in the CON group (*P* < 0.05).

## Discussion

Oxidative stress is one of the critical factors leading to growth retardation and disruption of various tissues and organs in neonatal mammals (3). Accumulating evidences showed that the intestine was more susceptible to oxidative stress than other tissues because of the frequent renewal of enterocytes and continuous exposure to various stimuli from the intestinal tract (4, 5). Oxidative stress could lead to overproduction of ROS, which would damage the integrity of intestinal barrier by disrupting the tight junction and triggering apoptosis of intestinal epithelium (15, 17). Previous research indicated that pigs subjected to oxidative stress usually showed negative growth performance (18). Similarly, in the present study, intraperitoneal injection of diquat significantly decreased the ADFI and ADG in weaned pigs. However, dietary CGA supplementation improved the growth performance in the diquat-challenged weaned pigs, which is consistent with previous study (19), indicating that dietary CGA supplementation could alleviate the growth retardation of weaned pigs upon oxidative stress.

Diamine oxidase, an intracellular enzyme, is mainly synthesized and distributed in the intestinal epithelium (20). Moreover, D-lactate is the special end product of bacteria in intestinal epithelia which exists mainly in cytoplasm. Once the integrity of intestinal barrier is destroyed, the levels of serum DAO and D-lactate will increase (16). Therefore, the DAO activity and D-lactate level in serum are commonly adopted to monitor the injury degree of the intestinal barrier. In the present study, diquat-challenged pigs exhibited an increase in the serum levels of DAO and D-lactate, indicating that the intestinal barrier function was impaired and intestinal oxidative damage model was successfully constructed. Inversely, CGA significantly alleviated these adverse effects induced by diquat, which was in line with the previous results that dietary CGA supplementation effectively decreased the serum DAO activity in lipopolysaccharide (LPS)-challenged weaned rats (21), demonstrating that dietary CGA supplementation helped to maintain the intestinal integrity of weaned pigs under oxidative stress.

Oxidative stress, a consequence of the imbalance of redox status, is closely related to animal health. GSH-Px and CAT are two critical antioxidant enzymes responsible for elimination of organic hydroperoxides and hydrogen peroxide (22), and MDA is widely considered to be an index used to monitor the degree of lipid peroxidation in mammals (23). Therefore, their serum levels have been widely used as biomarkers for monitoring the antioxidant capacity of the body. Present study showed that supplementation of dietary CGA could attenuate the negative effects of diquat challenge on activities of GSH-Px and SOD in weaned pigs and suppress the diquat-induced increase in the MDA concentration in serum. Similarly, a previous study in rat showed that CGA supplementation had protective effect against methotrexate-induced liver oxidative injury through increasing glutathione (GSH) activity and decreasing MDA concentration (9). A recent study in porcine jejunum epithelial cell line (IPEC-J2) cells also demonstrated that CGA could attenuate diquat-induced intestinal oxidative injury, which was associated with elevated antioxidant capacity (24). Therefore, CGA could maintain the antioxidant capacity of weaned pigs by improving the antioxidant enzymes activities. In addition, the antioxidant property of CGA may partly result from its special chemical structure. The CGA contains five hydroxyl groups, allowing itself to have the ability to scavenge the free radical (12).

Oxidative stress-induced overproduction of ROS was reported to cause intestinal morphological impairment (25). The integrity of intestinal villus-crypt morphological structure acts as the basis of nutrient digestion and absorption and is critical for maintaining the intestinal barrier function (26). The villus extends into the lumen to increase the surface area available for nutrition absorption, and the crypt is formed by the physically protected epithelial invagination that surrounds the villus base (27). In the current study, CGA administration significantly alleviated diquat-induced villus atrophy and crypt hyperplasia in jejunum of weaned pigs, suggesting that dietary CGA supplementation could improve the intestinal development of pigs upon oxidative stress possibly by the enhancing intestinal mucosal morphology integrity. Intestinal epithelium is the most important site for nutrient digestion and absorption, in which a variety of enzymes are expressed and secreted by the enterocytes (2). Obviously, disruption of the intestinal epithelium will result in a decrease of the enzyme activities in the intestinal mucosa. Previous study has shown that diquat-induced oxidative stress could cause serious dysfunctions of nutrient digestion and absorption in weaned pigs (14). Consistently, the results of the current study indicated that intraperitoneal injection of diquat significantly decreased the mucosal activities of disaccharidases (sucrase, lactase, and maltase) in the duodenum and jejunum and suppressed the activity of AKP in the jejunum and ileum of weaned pigs. However, CGA supplementation significantly alleviated the negative effects of diquat administration on intestinal mucosal enzyme activities, indicating the protective effects of CGA on digestion and absorption function of intestine in weaned pigs under oxidative stress. In addition, oxidative stress was also found to affect the expressions of nutrient transporters, leading to a decrease in nutrient absorption capacity (14, 15). Consistently, we further detected the mRNA expression of SGLT1 and GLUT2, which are critical glucose transporters located in the mucosa of the small intestine (28). The results showed that supplementation with CGA significantly suppressed the reduction of SGLT1 and GLUT2 expression levels induced by oxidative stress, indicating an increase energy supply for the growth of diquat-challenged weaned pigs. These results suggested that CGA supplementation was helpful in improving the intestinal digestion and absorption function of weaned pigs under oxidative stress, which may partly account for the promoting effect of CGA on growth performance as mentioned above (14).

Previous study reported that intraperitoneal injection of diquat could lead to the imbalance of redox status and then destroy the intestinal barrier function by influencing the expression of key molecules (15). Therefore, we further quantified the expression levels of critical genes related to the intestinal barrier functions. The intestinal barrier is mainly composed of the intercellular tight junction proteins, including transmembrane proteins (e.g., occludin and claudin families), intracellular linker proteins (ZOs), and many other regulatory proteins (29). Thus, tight junction proteins are essential for the integrity of the intestinal barrier by regulating the paracellular permeability. As expected, the present study showed that diquat challenge decreased the expression levels of occludin and claudin-1 in the duodenum and jejunum, which was consistent with a previous study that the increased intestinal permeability of pigs was associated with downregulation of tight junction proteins upon oxidative stress (30). Notably, CGA supplementation enhanced their expression levels in the intestinal mucosa. These results indicated that dietary CGA supplementation could alleviate oxidative stress-induced intestinal barrier dysfunction, which was associated with the improvement of intercellular junctions between epithelial cells. In addition, a previous study suggested that the oxidative stress-induced atrophy of intestinal villus and barrier dysfunction were partially associated with the apoptosis of intestinal epithelial cells (31). It is a well-known fact that the cell apoptosis is mainly regulated by multiple molecules, especially by the Bcl-2 and caspase family (32). Oxidative stress was found to promote the expression of a series of apoptosis-related genes (30). In this study, diquat challenge significantly elevated the expression levels of the Bax, caspase-3, and caspase-9 in the duodenal and jejunal mucosa. Interestingly, CGA significantly decreased the Bax, caspase-3, and caspase-9 expression levels in small intestinal mucosa, which was probably due to the elevated antioxidant capacity of pigs in DCGA group. Therefore, these results suggested that CGA could improve the intestinal barrier integrity partly by maintaining the tight junction protein expression and suppressing the excessive apoptosis of intestinal epithelial cells in weaned pigs under oxidative stress.

The elevated antioxidative capacity was also supported by the expressions of several critical antioxidant genes. Nrf2, as a master regulator of the antioxidant response, has been implicated in regulating the expression levels of endogenous antioxidant enzymes that protect against oxidative stress (33). It has been reported that the activities of antioxidant enzymes could be enhanced by increasing the mRNA expression levels of Nrf2 (34). Similarly, we found that CGA significantly elevated the expression levels of Nrf2 in the diquat-challenged weaned pigs in this study, which was in line with the increased activities of antioxidant enzymes mentioned above. A previous study in mice has suggested that polyphenols could further upregulate the expression of many antioxidative and cytoprotective genes by activating Nrf2 in the small intestine (35). HO-1 is one of the key antioxidant enzymes, which is located downstream of the Nrf2 and plays an important role in regulating the ROS levels of cells challenged by various stress (36). In this study, CGA supplementation significantly elevated the expression level of HO-1 in diquat-challenged pigs, which was in accord with the results of previous study (36), further indicating that CGA supplementation could enhance the antioxidant capacity in oxidative stress-challenged weaned pigs.

## Conclusion

In conclusion, our results showed that oxidative stress not only decreased the growth performance but also induced disruption of the intestinal barrier in the weaned pigs. Dietary CGA supplementation could attenuate the oxidative stress-induced growth retardation and intestinal barrier disruption, which was associated with elevated antioxidant capacity and improved intestinal barrier integrity.

## Data Availability Statement

The original contributions presented in the study are included in the article/Supplementary Material, further inquiries can be directed to the corresponding author.

## Ethics Statement

The animal study was reviewed and approved by the Animal Care and Use Committee of Sichuan Agricultural University (SICAU-2015-034).

## Author Contributions

JC and JH designed the experiment and wrote the manuscript. JC, DC, and BY performed the statistical analyses. YL, PZ, and XM carried out the animal experiment. JY and JL conducted the sample collection. ZH and HY carried out the sample analysis. All authors read and approved the final manuscript.

## Funding

This work was supported by the National Natural Science Foundation of China (31972599) and the Key Developmental Program of Sichuan Province (20ZDYF0003).

## Conflict of Interest

The authors declare that the research was conducted in the absence of any commercial or financial relationships that could be construed as a potential conflict of interest.

## Publisher's Note

All claims expressed in this article are solely those of the authors and do not necessarily represent those of their affiliated organizations, or those of the publisher, the editors and the reviewers. Any product that may be evaluated in this article, or claim that may be made by its manufacturer, is not guaranteed or endorsed by the publisher.
