# Supplementary material for: Chlorogenic Acid Attenuates Oxidative Stress-Induced Intestinal Mucosa Disruption in Weaned Pigs
Source: Front Vet Sci. 2022 Feb 14;9:806253. doi: 10.3389/fvets.2022.806253 (PMC8884245; doi:10.3389/fvets.2022.806253)
Supplement: Supplementary file 1 [file Data_Sheet_1.docx]

Supplementary Material

**Supplementary Table S1** **|** Ingredient composition and nutrient levels of basal diets (air-dry basis, %)

| Ingredient | % | Nutrient concentrations^1^ | % |
| --- | --- | --- | --- |
| Corn | 28.00 | CP | 20.36 |
| Extruded corn | 28.00 | ME (MJ/kg) | 14.83 |
| Soybean meal | 10.00 | Ca | 0.82 |
| Extruded soybean | 7.00 | Total P | 0.61 |
| Fish meal | 5.00 | Available P | 0.43 |
| Whey powder | 7.00 | Lysine | 1.37 |
| Soybean protein concentrate | 8.00 | Methionine | 0.45 |
| Soybean oil | 2.16 | Methionine + cystine | 0.74 |
| Sucrose | 2.50 | Threonine | 0.81 |
| Limestone | 0.70 | Tryptophan | 0.21 |
| Dicalcium phosphate | 0.45 |  |  |
| Salt | 0.30 |  |  |
| L-lysine HCl | 0.28 |  |  |
| DL-Methionine | 0.12 |  |  |
| L-Threonine | 0.04 |  |  |
| Choline chloride | 0.10 |  |  |
| Vitamin premix^2^ | 0.05 |  |  |
| Mineral premix^3^ | 0.30 |  |  |

^1^Values are calculated

^2^The premix provides following per kilogram of diet: Vitamin A, 6000 IU; Vitamin D_3,_ 400 IU; Vitamin E, 10 IU; Vitamin K_3,_ 2 mg; Vitamin B_1,_ 0.8 mg; Vitamin B_2,_ 6.4 mg; Vitamin B_6,_ 2.4 mg; Vitamin B_12,_ 12 µg; folic acid, 0.2 mg; nicotinic acid, 14 mg; D-pantothenic acid, 10 mg

^3^The premix provides following per kilogram of diet: Fe (as ferrous sulfate), 130 mg; Cu (as copper sulfate), 80 mg; Mn (as manganese sulfate), 60 mg; Zn (zinc sulfate), 120 mg; I (potassium iodide), 0.3 mg; Se (as sodium selenite), 0.35 mg

**Supplementary Table S2** **|** Primers used for real-time quantitative PCR^1^.

| Gene | Accession NO. | Primer sequences^2^ (5'-3') | Size,bp |
| --- | --- | --- | --- |
| SGLT1 | NM_001164021.1 | F: GCAACAGCAAAGAGGAGCGTAT | 137 |
|  |  | R: GCCACAAAACAGGTCATAGGTC |  |
| GLUT2 | [NM_001097417.1](https://www.ncbi.nlm.nih.gov/entrez/viewer.fcgi?db=nucleotide&id=47523065) | F: GACACGTTTTGGGTGTTCCG | 149 |
|  |  | R: GAGGCTAGCAGATGCCGTAG |  |
| ZO-1 | XM-003480423.4 | F: CAGAGACCAAGAGCCGTCC | 105 |
|  |  | R: TGCTTCAAGACATGGTTGGC |  |
| occludin | NM-001163647.2 | F: TCAGGTGCACCCTCCAGATT | 118 |
|  |  | R: AGGAGGTGGACTTTCAAGAGG |  |
| claudin-1 | [NM-001244539.1](https://www.ncbi.nlm.nih.gov/entrez/viewer.fcgi?db=nucleotide&id=1191888577) | F: ATTTCAGGTCTGGCTATCTTAGTTGC | 214 |
|  |  | R: AGGGCCTTGGTGTTGGGTAA |  |
| Bax | XM_ 013998624.2 | F: GACGCTGGACTTCCTTCGAG | 334 |
|  |  | R: GTGGCCCGAGAGAGGTTTATT |  |
| Bcl-2 | XM_021099593.1 | F: GCTACTTACTGCCAAAGGGA | 161 |
|  |  | R: TTCAGGCGGAGCTGTAAGAG |  |
| Caspase-3 | NM_214131.1 | F: GGAATGGCATGTCGATCTGGT | 351 |
|  |  | R: ACTGTCCGTCTCAATCCCAC |  |
| Caspase-9 | XM_013998997.2 | F: AATGCCGATTTGGCTTACGT | 195 |
|  |  | R: CATTTGCTTGGCAGTCAGGTT |  |
| Nrf2 | XM_021075133.1 | F: GCCCCTGGAAGCGTTAAAC | 67 |
|  |  | R: GGACTGTATCCCCAGAAGGTTGT |  |
| Keap1 | XM_021076667.1 | F: ACGACGTGGAGACAGAAACGT | 56 |
|  |  | R: GCTTCGCCGATGCTTCA |  |
| HO-1 | NM_001004027.1 | F: AGCTGTTTCTGAGCCTCCAA | 130 |
|  |  | R: CAAGACGGAAACACGAGACA |  |
| GAPDH | NM_001206359.1 | F: TCGGAGTGAACGGATTTGGC | 147 |
|  |  | R: TGCCGTGGGTGGAATCATAC |  |

^1^ SGLT1, sodium glucose transport protein-1; GLUT2, glucose transporter-2; ZO-1, zonula occludens 1; Bax, B-cell lymphoma-2-associated X protein; Bcl-2, B-cell lymphoma-2; Nrf2, nuclear factor erythroid-derived 2-related factor 2; Keap1, kelch-like epichlorohydrin-associated protein 1; HO-1, heme oxygenase-1; GAPDH, glyceraldehyde-3-phosphate dehydrogenase.

^2^F, forward, R, reverse.

**
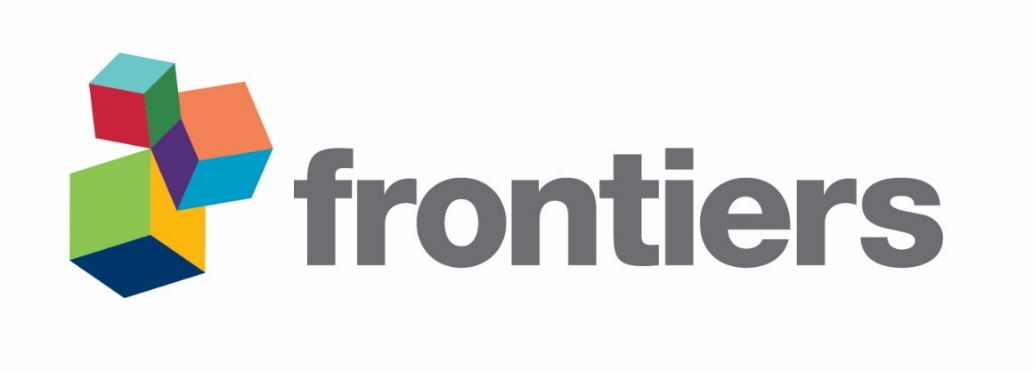
**
